# Watching Videos and Television Is Related to a Lower Development of Complex Language Comprehension in Young Children with Autism

**DOI:** 10.3390/healthcare9040423

**Published:** 2021-04-06

**Authors:** Elisabeth Fridberg, Edward Khokhlovich, Andrey Vyshedskiy

**Affiliations:** 1 Biology Department, Boston University, Boston, MA 02215, USA; liza.fridberg@gmail.com; 2 ImagiRation, Boston, MA 02135, USA; 3 Independent Researcher, Newton, MA 02459, USA; edpiter@gmail.com

**Keywords:** autism, ASD, language delay, screen time, television time, receptive language, language comprehension

## Abstract

The effect of passive video and television watching duration on 2- to 5-year-old children with autism was investigated in the largest and the longest observational study to date. Parents assessed the development of 3227 children quarterly for three years. Longer video and television watching were associated with better development of expressive language but significantly impeded development of complex language comprehension. On an annualized basis, low TV users (low quartile: 40 min or less of videos and television per day) improved their language comprehension 1.4 times faster than high TV users (high quartile: 2 h or more of videos and television per day). This difference was statistically significant. At the same time, high TV users improved their expressive language 1.3 times faster than low TV users. This difference was not statistically significant. No effect of video and television watching duration on sociability, cognition, or health was detected.

## 1. Introduction

High exposure to TV has been found to negatively affect language acquisition in young neurotypical children. Chonchaiya et al. showed that children younger than one year who watched more than 2 h of TV per day were six times more likely to have language delays than their peers who watched less than 1 h per day [1]. In a parent-reported investigation of 2441 children aged 2 and 3, Madigan et al. demonstrated that high screen use was associated with delayed development measured one and two years later [2]. Zimmerman et al. used a parent-reported measure to assess language development in 1008 children between the ages of 2 months and 24 months. Exposure to television led to a 28% decrease in the score for every hour of viewing in the 8–16-month age group [3]. Byeon et al. surveyed the parents of 1778 toddlers to find that the risk of language delay increased proportionally with TV watching time. Children that watched 3 h of TV per day were three times more likely to develop a language delay than their counterparts that watched less than 1 h per day [4]. In addition to language delay, high TV exposure has been implicated in decreased executive functioning, lower cognitive abilities, reduced short-term memory, increases in rates of anxiety and depression, as well as reduced emotional stability, and self-control [5,6,7,8,9]. Thus, there is strong consensus that passive TV and video watching has a negative effect on young children, and the American Academy of Pediatrics recommends that television and video time be kept to a minimum in children 2 years and older, and avoided in younger children [10].

In contrast to neurotypical children, studies of television use in children diagnosed with Autism Spectrum Disorder (ASD) are often framed to uphold the benefits of television. One such study touted the benefits of TV on vocabulary [11]. Another study suggested the benefits on social development [12]. While increased vocabulary and social modeling may have a significant positive effect on ASD children’s quality of life, it is important to consider the potentially harmful effects of television on their overall language development. This begs the question of whether ASD children, who have a unique developmental trajectory, are similarly impacted by early exposure to television. As many children with ASD have language deficits, it is integral to define the role of television exposure on their symptoms.

Our unique position of operating a popular language therapy app provided us with thousands of parent-reported evaluations of children with ASD collected longitudinally. Through obligatory quarterly assessments, we also collected data on video and television watching habits of children enrolled in the program. In order to identify the effect of video and television, we compared high and low television users on five orthogonal measures: (1) receptive language, (2) expressive language, (3) sociability, (4) cognitive awareness, and (5) health.

For the purposes of our research, receptive language was defined as complex language that uses modifiers, spatial prepositions, and fictitious situations, which require visuospatial mental integration rather than memorization. Complex language comprehension is illustrated by the two phrases “cat on a mat” and “mat on a cat”. While the two phrases share the same vocabulary and the same grammar, a neurotypical individual can easily tell the difference. When reading the two phrases, one visualizes the two situations in their mind’s eye and places the image of the cat on top of the image of the mat. Similarly, if the phrase was modified to become “big black cat on a tiny wet mat”, one would immediately adjust the mental image to reflect the additional details. When the new phrase is rearranged to “big black mat on a wet cat”, the same process of disassembly and reassembly takes place. Thus, a completely new image is constructed, despite the fact that most people have never seen a mat on a cat. This ability to combine and recombine novel mental images at will (called prefrontal synthesis or PFS [13]) is essential for complex receptive language, including comprehension of spatial prepositions, semantically reversible sentences, and recursion [14,15].

## 2. Methods

### 2.1. Participants

Participants were users of a language therapy app that was made available gratis at all major app stores in September 2015. Once the app was downloaded, caregivers were asked to register and to provide demographic details, including the child’s diagnosis and age. Caregivers consented to anonymized data analysis and completed the Autism Treatment Evaluation Checklist (ATEC) [16], an evaluation of the receptive language using the Mental Synthesis Evaluation Checklist (MSEC) [17], as well as the Screen Time assessment. The first evaluation was administered approximately one month after the download. The subsequent evaluations were administered at approximately three-month intervals. To enforce regular evaluations, the app became unusable at the end of each three-month interval, and parents were required to complete an evaluation to regain its functionality.

### 2.2. Inclusion Criteria

From this pool of potential study participants, we selected participants based on the following criteria:

(1) Consistency: Participants must have filled out at least three ATEC evaluations and the interval between the first and the last evaluation was 6 months or longer.
(2) Diagnosis: ASD. Children without ASD diagnosis were excluded from the study. The parent-reported ASD diagnosis was not verified directly, as we cannot ask participants to submit documentation. However, ATEC scores support ASD diagnosis. Average initial ATEC total score was 74.9 ± 24.0, which corresponds to moderate-to-severe ASD as delineated in [18] and Table 1.

### 2.3. Exclusion Criteria

(1) Maximum age: Participants older than five years of age were excluded from this study.
(2) Minimum age: Participants who completed their first evaluation before the age of two years were excluded from this study.

After excluding participants that did not meet these criteria, there were 3227 total participants.

### 2.4. Evaluations

A caregiver-completed Autism Treatment Evaluation Checklist (ATEC) [16] and Mental Synthesis Evaluation Checklist (MSEC) [17] were used to track child development. The ATEC questionnaire comprises four subscales: (1) speech/language/communication, (2) sociability, (3) sensory/cognitive awareness, and (4) physical/health/behavior. The first subscale, speech/language/communication, contains 14 items, and its score ranges from 0 to 28 points. The sociability subscale contains 20 items within a score range of 0 to 40 points. The third subscale, referred to here as the cognitive awareness subscale, has 18 items, and scores range from 0 to 36 points. The fourth subscale, referred to here as the health subscale, contains 25 items, and scores range from 0 to 75 points. The scores from each subscale are combined in order to calculate a total score, which ranges from 0 to 179 points. A lower score indicates lower severity of ASD symptoms, and a higher score indicates more severe symptoms of ASD. ATEC is not a diagnostic checklist. It was designed to evaluate the effectiveness of treatment [16]; thus, ASD severity can only have an approximate relationship with the total ATEC score and age. Table 1 lists approximate ATEC total score as related to ASD severity and age as described in Mahapatra et al. [19].

ATEC was selected because it is one of the few measures validated to evaluate treatment effectiveness. In contrast, another popular ASD assessment tool, Autism Diagnostic Observation Schedule or ADOS [20], has only been validated as a diagnostic tool. Various studies have confirmed the validity and reliability of ATEC [21,22,23], and several trials confirmed ATEC’s ability to longitudinally measure changes in participant performance [19,24,25,26]. Whitehouse et al. used ATEC as a primary outcome measure for a randomized controlled trial of their iPad-based intervention for ASD, named “Therapy Outcomes By You” or TOBY, and noted ATEC’s “internal consistency and adequate predictive validity” [27]. These studies support the effectiveness of ATEC as a tool for longitudinal tracking of symptoms and assessing changes in ASD severity.

### 2.5. Expressive Language Assessment

The ATEC speech/language/communication subscale includes the following questions: (1) knows own name, (2) responds to “No” or “Stop”, (3) can follow some commands, (4) can use one word at a time (No!, Eat, Water, etc.), (5) can use two words at a time (Don’t want, Go home), (6) can use three words at a time (Want more milk), (7) knows 10 or more words, (8) can use sentences with four or more words, (9) explains what they want, (10) asks meaningful questions, (11) speech tends to be meaningful/relevant, (12) often uses several successive sentences, (13) carries on fairly good conversation, and (14) has normal ability to communicate for their age. With the exception of the first three items, all items in the ATEC subscale 1 primarily measure expressive language. Accordingly, the ATEC subscale 1 is referred to in this manuscript as the expressive language subscale to distinguish it from the receptive language subscale tested by the MSEC evaluation.

### 2.6. Receptive Language Assessment

The MSEC evaluation was designed to be complementary to ATEC in measuring complex receptive language. Out of 20 MSEC items, those that directly assess receptive language are the following: (1) understands simple stories that are read aloud, (2) understands elaborate fairy tales that are read aloud (i.e., stories describing FANTASY creatures), (6) understands some simple modifiers (i.e., green apple vs. red apple or big apple vs. small apple), (7) understands several modifiers in a sentence (i.e., small green apple), (8) understands size (can select the largest/smallest object out of a collection of objects), (9) understands possessive pronouns (i.e., your apple vs. her apple), (10) understands spatial prepositions (i.e., put the apple ON TOP of the box vs. INSIDE the box vs. BEHIND the box), (11) understands verb tenses (i.e., I will eat an apple vs. I ate an apple), (12) understands the change in meaning when the order of words is changed (i.e., understands the difference between “a cat ate a mouse” vs. “a mouse ate a cat”), and (20) understands explanations about people, objects or situations beyond the immediate surroundings (e.g., “Mom is walking the dog”, “The snow has turned to water”). MSEC consists of 20 questions within a score range of 0 to 40 points; similarly to ATEC, a lower score indicates better receptive language.

The psychometric quality of MSEC was tested with 3715 parents of ASD children [17]. Internal reliability of MSEC was good (Cronbach’s alpha > 0.9). MSEC exhibited adequate test–retest reliability, good construct validity, and good known group validity reflected by the difference in MSEC scores for children of different ASD severity levels.

To simplify interpretation of figure labels, the subscale 1 of the ATEC evaluation is referred to here as the expressive language subscale and the MSEC scale is referred to as the receptive language subscale.

### 2.7. Video and Television Watching Time Assessment

In addition to the ATEC and MSEC evaluations, participants were required to respond to the question: “How much time does your child spend watching any videos? (YouTube, TV) (each day)”. To assess the effect of video and television watching time, we compared participants in the high-video-watching-duration quartile (high TV users) to participants in the low-video-watching-duration quartile (low TV users), Table 2. The high TV users were matched to the low TV users by age, gender, expressive language, receptive language, sociability, cognitive awareness, and health at the 1st evaluation (baseline) using propensity score analysis [28].

### 2.8. Statistical Analysis

The framework for the evaluation of score changes over time was explained in detail in Mahapatra et al. [19] and Vyshedskiy et al. [14]. In short, the concept of a “Visit” was developed by dividing the 3-year-long observation interval into 3-month periods. All evaluations were mapped into 3-month-long bins with the first evaluation placed in the first bin. When more than one evaluation was completed within a bin, their results were averaged to calculate a single number representing this 3-month interval. Thus, we had 12 quarterly evaluations for both high- and low-TV-duration groups.

It was then hypothesized that there was a two-way interaction between TV duration group and Visit. Statistically, this hypothesis was modeled by applying the Linear Mixed Effect Model with Repeated Measures (MMRM), where a two-way interaction term was introduced to test the hypothesis. The model (Endpoint~Baseline + Gender + Severity + TV-duration-group * Visit) was fit using the R Bioconductor library of statistical packages, specifically the “nlme” package. The subscale scores at baseline, gender, and severity were used as covariates. Conceptually, the model fits a plane into n-dimensional space. This plane considers a complex variability structure across multiple visits, including baseline differences. Once such a plane is fit, the model calculates Least Squares Means (LS Means) for each subscale and TV duration group at each visit. The model also calculates LS Mean differences between the high-TV-duration group and low-TV-duration group at each visit. Participants in the high-TV-duration group were matched to those in the low-TV-duration group using propensity score analysis [28] based on age, gender, expressive language, receptive language, sociability, cognitive awareness, and health at the 1st evaluation (baseline).

### 2.9. Informed Consent

Caregivers consented to anonymized data analysis and publication of the results. The study was conducted in compliance with the Declaration of Helsinki [29].

### 2.10. Compliance with Ethical Standards

Using the Department of Health and Human Services regulations found at 45 CFR 46.101 (b) (1), it was determined that this research project is exempt from institutional review board oversight.

### 2.11. Data Availability

De-identified raw data from this manuscript are available from the corresponding author upon reasonable request.

### 2.12. Code Availability Statement

Code is available from the corresponding author upon reasonable request.

## 3. Results

**Figure 1:**
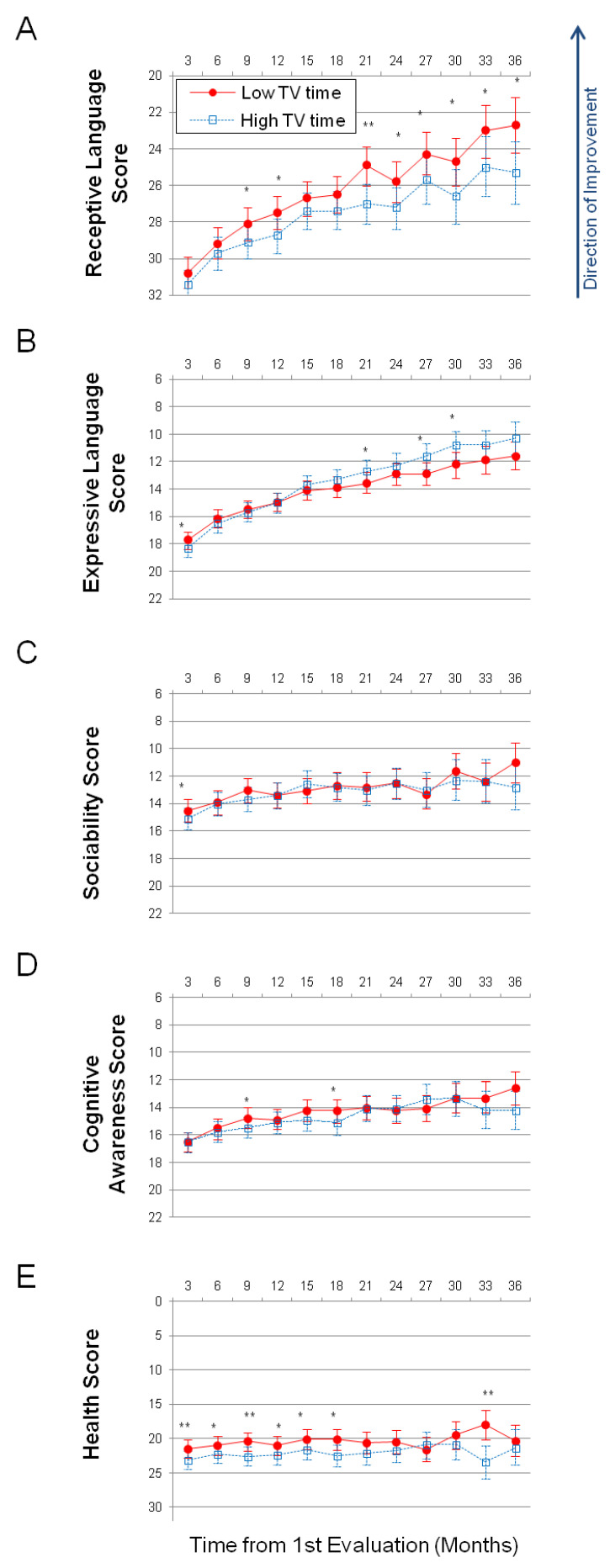
Longitudinal plots of subscale scores LS Means. Horizontal axis shows months from the 1st evaluation (0 to 36 months). Error bars show the 95% confidence interval. To facilitate comparison between subscales, all vertical axes ranges have been normalized to show 40% of their corresponding subscale’s maximum available score. A lower score indicates symptom improvement. P-value is marked: ** *p* < 0.001; * *p* < 0.05. (**A**) Receptive language score. (**B**) Expressive language score. (**C**) Sociability score. (**D**) Cognitive awareness score. (**E**) Health score.

On the receptive language subscale, the average improvement in high TV users over 36 months was 6.09 points (SE = 0.80, *p* < 0.0001) compared to 8.08 points (SE = 0.68, *p* < 0.0001) in low TV users, Figure 1A, Table 3, Table S1. The difference in high TV users relative to low TV users at month 36 was statistically significant: 2.58 points (SE = 1.04, *p* = 0.0128). The positive difference (marked in the Table 3 as “High–Low”) indicates that high TV users had greater scores at month 36 and, therefore, more severe symptoms. On an annualized basis, low TV users improved their receptive language 1.4 times faster than high TV users (high TV users = 2.0 points/year; low TV users = 2.7 points/year).

Conversely, on the expressive language subscale, high TV users improved their score to a greater extent than low TV users. High TV users improved over the 36-month period by 7.96 points (SE = 0.54, *p* < 0.0001) compared to 6.17 points (SE = 0.45, *p* < 0.0001) improvement in low TV users, Figure 1B, Table S2. The difference in high TV users relative to low TV users at month 36 was not statistically significant: −1.26 points (SE = 0.7, *p* = 0.0719). On an annualized basis, high TV users improved their expressive language 1.3 times faster than low TV users (high TV users = 2.7 points/year; low TV users = 2.1 points/year).

On the sociability subscale, high TV users improved over the 36-month period by 2.27 points (SE = 0.77, *p* = 0.0032) compared to 3.46 points (SE = 0.65, *p* < 0.0001) improvement in low TV users, Figure 1C, Table S3. The difference in high TV users relative to low TV users at month 36 was not statistically significant: 1.82 points (SE = 0.99, *p* = 0.0663).

On the cognitive awareness subscale, high TV users improved over the 36-month period by 2.26 points (SE = 0.66, *p* = 0.0006) compared to 3.89 points (SE = 0.56, *p* < 0.0001) improvement in low TV users, Figure 1D, Table S4. The difference in high TV users relative to low TV users at month 36 was not statistically significant: 1.58 points (SE = 0.85, *p* = 0.0631).

On the health subscale, high TV users improved over the 36-month period by 1.78 points (SE = 1.18, *p* = 0.1308) compared to 1.19 points (SE = 0.99, *p* = 0.2296) improvement in low TV users, Figure 1E, Table S5. The difference in high TV users relative to low TV users at month 36 was not statistically significant: 1.05 points (SE = 1.52, *p* = 0.4898).

## 4. Discussion

The effect of watching videos and television is a subject of controversy for children with Autism Spectrum Disorder (ASD). Some investigators uphold the benefits of video and television learning for ASD children [30], while others urge caretakers to consider the potential negative effects of screen exposure observed in neurotypical children [1]. In the past, videos and television were heralded as beneficial for children with ASD due to the related increase in vocabulary in some children [11]. However, the effect of videos and television on complex language comprehension has never been investigated.

In order to evaluate complex language comprehension in children with ASD, our study provided parents with a questionnaire called MSEC. MSEC is composed of 20 language comprehension items that steadily increase in difficulty. The items evaluate comprehension of simple and compound modifiers (such as color and size), spatial prepositions, possessive pronouns, verb tense, and semantically reversible sentences. Another aspect of MSEC focuses on fairytale comprehension. As fairytales require the listener to imagine unrealistic situations, they are a good indicator of complex language comprehension.

In addition to MSEC, parents completed the Autism Treatment Evaluation Checklist (ATEC) [16], which reports a score over four orthogonal subscales: expressive language, sociability, cognitive awareness, and health. Parents used MSEC and ATEC to assess the development of 3227 children quarterly for up to three years, making this the largest and the longest study of the effect of video and television watching on children with ASD to date. Children in the high-TV-watching-duration quartile (high TV users) watched videos and television for 2 or more hours per day. Children in the low-TV-watching-duration quartile (low TV users) watched videos and television for 40 min or less per day. No significant effect of video and television watching duration on sociability, cognition, or health was detected. High TV users demonstrated 1.3 times faster development of expressive language, although the difference between high TV and low TV users was not statistically significant at month 36 (the difference was statistically significant at months 21, 27, and 30). The greatest difference was found in the MSEC scale. Low TV users demonstrated significantly greater development of receptive language. The difference between high TV and low TV users was statistically significant in all evaluations completed during the 2nd half of the study (months 21, 24, 27, 30, 33, and 36). On an annualized basis, low TV users improved their receptive language 1.4 times faster than high TV users (Figure 1; low TV users = 2.7 points/year; high TV users = 2.0 points/year). The apparent difference in developmental trajectories suggests that excessive watching of videos and television can diminish the ability of young ASD children to reach their full potential in regard to complex language comprehension.

The video and television duration assessment question read “How much time does your child spend watching any videos? (YouTube, TV) (each day)” and clearly excluded educational apps. The parent-submitted evaluation included two other questions about educational apps that preceded the TV duration question: “How much time does your child spend using any educational apps under adult supervision? (each day)” and “How much time does your child spend using any educational apps on his own? (each day)”. Analysis of educational app use duration showed no difference between the high- and low-use quartiles in receptive language, expressive language, sociability, and cognitive awareness (data not shown). Thus, our results show that only passive video and television watching impede acquisition of complex language comprehension, confirming the common adage that “all screen time is not created equal”.

### 4.1. Limitations

The observational design of this study cannot definitively prove causality, since unknown confounders may influence the study results. However, the golden standard for testing causation—a randomized controlled trial (RCT)—is not feasible due to obvious ethical concerns. One popular and increasingly common alternative to RCT is called the propensity score analysis [28]. The propensity score analysis is used to identify comparable individuals in observational study cohorts [31,32]. This study utilized the propensity score analysis to match high TV participants to low TV participants based on age, gender, expressive language, receptive language, sociability, cognitive awareness, and health score at the 1st evaluation (baseline).

The data used in this study were collected from parents using a language therapy app. The fact that parents supplemented their children’s language therapy with additional exercises provided by the language therapy app over several years argues for significant self-motivation and attention to their children’s language therapy. In less motivated families, the effect of videos and television can be even greater.

Socioeconomic status data were not collected. However, if the socioeconomic status was the factor generating the improvement in the receptive language score in the low TV users, then we would expect a similar improvement in the expressive language score. Conversely, expressive language developed better in the high TV users. This dissociation between receptive and expressive language trajectories suggests that rather than being an effect of socioeconomic status, passive video and television watching itself reduced children’s complex language comprehension. By spending significant time passively watching videos and television, children were most likely deprived of the active top-down mental stimulation provided by fairy tales and complex recursive conversations with their caregivers, which are known to be important in language acquisition [33].

A similar argument can be used to reject the language-therapy-duration confounder. If low TV users received a greater duration of language therapy, they would be expected to display greater improvement on both the receptive and expressive language subscales. The dissociation of the receptive and expressive language trajectories makes the language therapy confounding highly unlikely. Furthermore, adding the duration of language therapy as a covariate to the Linear Mixed Effect Model with Repeated Measures demonstrated that the duration of language therapy covariate was not statistically significant in either the receptive language subscale (*p* = 0.5366) or the expressive language subscale (*p* = 0.4708).

An evaluation bias confounder can also be rejected. If one group of parents was more likely to rate their children as improving, they would be expected to consistently rate their children as improving across all subscales. The dissociation of the receptive and expressive language trajectories makes evaluation bias an unlikely explanation. In addition, there was little difference between high and low TV users’ trajectories on the sociability, cognitive awareness, or health subscales, making an evaluation bias even less likely.

### 4.2. Clinical Significance

The 2.58 point difference in receptive language observed between the low TV users and high TV users at the end of the study is clinically significant. At the average level of this study cohort (MSEC score = 22), the two-point disparity corresponds to the difference between a child understanding or not understanding spatial prepositions (i.e., parents answering ‘true’ or ‘not true’ on the question 10 of MSEC; every MSEC question is worth two points). The complete question 10 of MSEC reads: “Understands spatial prepositions (i.e., put the apple ON TOP of the box vs. INSIDE the box vs. BEHIND the box).” Naturally, understanding spatial prepositions at age 5 sets children on a different developmental trajectory compared to children who do not understand spatial prepositions.

Conversely, the −1.26 point difference in expressive language observed between the low TV users and high TV users at the end of the study is likely not clinically significant. At the average level of this study cohort (ATEC language subscale score = 12), the 1-point disparity corresponds to parents answering ‘not true’ or ‘somewhat true’ on the question 12: “Can use sentences with 4 or more words.” It is possible that the small gain in expressive language observed in high TV users is not functional, but merely imitative.

### 4.3. Implications

The process of combining and recombining novel mental images at will (prefrontal synthesis or PFS) is an essential mechanism of receptive language, necessary for understanding of spatial prepositions, semantically reversible sentences, and complex recursive language [13,14]. In many ways, combinatorial control of visuospatial mental objects by the prefrontal cortex is analogous to control of skeletal muscle movement by the motor cortex. In fact, PFS can be viewed as the evolutionary internalization of muscle movement.

The analogy between PFS and muscle movement extends to their development. Just like it is impossible to acquire muscle control from the passive watching of sports programs, it is equally impossible to develop PFS from the passive watching of cartoons and fairytales. Acquisition of fine motor control in children is an experience-dependent process; the experience is provided by the physical movement of muscles. Acquisition of PFS is also an experience-dependent process; the experience is primarily driven by the use of recursive language: through normal conversations, storytelling (internal or external), and reading fairy tales that require children to imagine unrealistic situations. Children who experience fewer conversations show a significant reduction of frontoposterior fiber tracts mediating PFS [33], and a complete lack of recursive conversations (in feral children and deaf linguistic isolates) is associated with lifelong PFS paralysis [34].

The results of this study complement existing evidence in neurotypical children: passive video and television watching does not develop PFS. Critically, passive video and television watching may be particularly detrimental for young children with ASD who may have a shorter critical period for PFS acquisition [35,36].

## 5. Conclusions

To our knowledge, this is the first comprehensive evaluation of video and television effect on both receptive and expressive language acquisition in children with ASD. This is also the largest and the longest study of the effect of video and television watching on the development of children with ASD. The study confirmed the previously reported positive effect of videos and television on expressive language but also revealed the previously unstudied negative effect of videos and television on children with ASD. After rejecting socioeconomic factors, language therapy duration, and evaluation bias confounders, our analysis led us to conclude that passive video and television watching itself impedes complex language comprehension in children with ASD. While our research shows significant language acquisition differences among children aged 2 to 5 years, future studies should assess the effect of videos and television on older and younger children, as well as children who are higher and lower on the autism spectrum.

In studying the effect of videos and television on language acquisition, we established a new epidemiological approach: in-app evaluation. In-app evaluation has proven to be an excellent way to study the longitudinal effect of different therapies in a large population of children. We anticipate its rise to prominence in the scientific and medical communities in the coming years due to its accessibility and ease of use.

Finally, the dissociation of the receptive and expressive language score trajectories shows the fallacy of defining language development only in terms of vocabulary (expressive language) but suggests the need for comprehensive language examination that includes complex receptive language [15].

## Figures and Tables

**Table 1:** Approximate relationship between Autism Treatment Evaluation Checklist (ATEC) total score, age, and Autism Spectrum Disorder (ASD) severity as described in Mahapatra et al. [19]. At any age, a greater ATEC score indicates greater ASD severity.

Severity	Age										
	2	3	4	5	6	7	8	9	10	11	12
Mild	<82	<65	<52	<43	<36	<31	<28	<25	<23	<21	<20
Moderate	82–130	65–103	52–83	43–69	36–58	31–50	28–44	25–39	23–36	21–34	20–32
Severe	130–179	103–179	83–179	69–179	58–179	50–179	44–179	39–179	36–179	34–179	32–179

**Table 2:** High- and low-video-watching-duration quartiles demographics data.

	High TV Users Quartile	Low TV Users Quartile
Video (TV, YouTube) viewing time	≥120 min/day	≤40 min/day
Number of participants	797	797
Age at baseline	3.4 ± 0.7	3.6 ± 0.8
Male Gender	71%	81%

**Table 3:** Characteristics of high and low TV user quartiles. Data are presented as Least Squares Means (LS Means) (SE; 95% CI). A lower score indicates a lower severity of ASD symptoms. The difference between high TV and low TV quartiles is presented as LS Mean (SE; *p*-value). The positive high–low difference indicates that high TV users had a higher score and therefore more severe symptoms.

Subscale	Baseline			Month 36			Month 36-Baseline	
	High TV Users	Low TV Users	High–Low	High TV Users	Low TV Users	High–Low	High TV Users	Low TV Users
Receptive Language	31.4 (0.43; 30.5–32.2)	30.8 (0.42; 29.9–31.6)	0.6 (0.3; 0.0505)	25.3 (0.88; 23.6–27)	22.7 (0.76; 21.2–24.2)	2.58 (1.04; 0.0128)	−6.09 (0.8; <0.0001)	−8.08 (0.68; <0.0001)
Expressive Language	18.3 (0.32; 17.64–18.9)	17.7 (0.32; 17.11–18.4)	0.53 (0.22; 0.0175)	10.3 (0.6; 9.12–11.5)	11.6 (0.53; 10.52–12.6)	−1.26 (0.7; 0.0719)	−7.96 (0.54; <0.0001)	−6.17 (0.45; <0.0001)
Sociability	15.1 (0.42; 14.29–15.9)	14.5 (0.41; 13.67–15.3)	0.63 (0.29; 0.0329)	12.8 (0.84; 11.19–14.5)	11 (0.74; 9.58–12.5)	1.82 (0.99; 0.0663)	−2.27 (0.77; 0.0032)	−3.46 (0.65; <0.0001)
Cognitive Awareness	16.5 (0.36; 15.7–17.2)	16.5 (0.35; 15.8–17.2)	−0.04 (0.25; 0.8737)	14.2 (0.72; 12.8–15.6)	12.6 (0.63; 11.4–13.8)	1.58 (0.85; 0.0631)	−2.26 (0.66; 0.0006)	−3.89 (0.56; <0.0001)
Health	23.1 (0.66; 21.8–24.4)	21.5 (0.66; 20.2–22.8)	1.63 (0.46; 0.0004)	21.3 (1.3; 18.8–23.9)	20.3 (1.14; 18–22.5)	1.05 (1.52; 0.4898)	−1.78 (1.18; 0.1308)	−1.19 (0.99; 0.2296)

## Data Availability

The data presented in this study are available on request from the corresponding author.

## Supplementary Materials

The following are available online at https://www.mdpi.com/article/10.3390/healthcare9040423/s1, Table S1: LS Means over visits for Receptive Language MSEC subscale score; Table S2: LS Means for Expressive Language measured by the Subscale 1 of ATEC; Table S3: LS Means for Sociability subscale score measured by the Subscale 2 of ATEC; Table S4: LS Means for the Sensory/Cognitive Awareness subscale score measured by the Subscale 3 of ATEC; Table S5: LS Means for Health/Physical/Behavior subscale score measured by the Subscale 4 of ATEC.
