# Supplementary material for: Watching Videos and Television Is Related to a Lower Development of Complex Language Comprehension in Young Children with Autism
Source: Healthcare (Basel). 2021 Apr 6;9(4):423. doi: 10.3390/healthcare9040423 (PMC8067341; doi:10.3390/healthcare9040423)
Supplement: Supplementary file 1 [file healthcare-09-00423-s001.pdf]

healthcare

MDPI

Supplementary Material

Watching Videos and Television is Related to a Lower Development of Complex Language Comprehension in Young Children with Autism

Elisabeth Fridberg, Edward Khokhlovich and Andrey Vyshedskiy

Table S1. LS Means (SE; 95% CI) for Receptive Language MSEC subscale score. The differences between High- and Low-duration quartiles and between Month 36 and Baseline are presented as: LS Mean (SE; P-value). A lower score indicates lower severity of ASD symptoms. The positive High-Low contrast indicates that the high-duration quartile had higher score and therefore more severe symptoms.

Visit Number	High-duration quartile	Low-duration quartile	High-Low
Baseline	31.4 (0.43; 30.5 - 32.2)	30.8 (0.42; 29.9 - 31.6)	0.6 (0.3; 0.0505)
Month 6	29.7 (0.45; 28.8 - 30.6)	29.2 (0.45; 28.3 - 30)	0.55 (0.37; 0.1363)
Month 9	29.1 (0.46; 28.2 - 30)	28.1 (0.45; 27.2 - 29)	1.03 (0.39; 0.008)
Month 12	28.7 (0.48; 27.7 - 29.6)	27.5 (0.47; 26.6 - 28.4)	1.22 (0.42; 0.004)
Month 15	27.4 (0.51; 26.4 - 28.4)	26.7 (0.49; 25.8 - 27.7)	0.67 (0.49; 0.1671)
Month 18	27.4 (0.54; 26.4 - 28.5)	26.5 (0.52; 25.5 - 27.5)	0.93 (0.54; 0.0835)
Month 21	27 (0.56; 25.9 - 28.1)	24.9 (0.54; 23.9 - 26)	2.11 (0.58; 0.0003)
Month 24	27.2 (0.6; 26 - 28.3)	25.8 (0.56; 24.7 - 26.9)	1.34 (0.63; 0.0328)
Month 27	25.7 (0.66; 24.4 - 27)	24.3 (0.58; 23.1 - 25.4)	1.45 (0.71; 0.0397)
Month 30	26.6 (0.77; 25.1 - 28.1)	24.7 (0.68; 23.4 - 26)	1.9 (0.88; 0.0304)
Month 33	25 (0.83; 23.4 - 26.7)	23 (0.73; 21.6 - 24.5)	1.99 (0.98; 0.0428)
Month 36	25.3 (0.88; 23.6 - 27)	22.7 (0.76; 21.2 - 24.2)	2.58 (1.04; 0.0128)
Month 36 - Baseline	-6.09 (0.8; <0.0001)	-8.08 (0.68; <0.0001)	na

Table S2. LS Means (SE; 95% CI) for Expressive Language measured by the Subscale 1 of ATEC. The differences between High- and Low-duration quartiles and between Month 36 and Baseline are presented as: LS Mean (SE; P-value). A lower score indicates lower severity of ASD symptoms. The negative High-Low contrast indicates that the high-duration quartile had lower score and therefore milder symptoms.

Visit Number	High-duration quartile	Low-duration quartile	High-Low
Baseline	18.3 (0.32; 17.64 - 18.9)	17.7 (0.32; 17.11 - 18.4)	0.53 (0.22; 0.0175)
Month 6	16.5 (0.33; 15.81 - 17.1)	16.2 (0.34; 15.5 - 16.8)	0.31 (0.26; 0.2451)
Month 9	15.7 (0.34; 15.05 - 16.4)	15.5 (0.34; 14.82 - 16.1)	0.24 (0.27; 0.3832)
Month 12	15 (0.35; 14.28 - 15.7)	15 (0.35; 14.28 - 15.6)	0.01 (0.3; 0.9736)
Month 15	13.7 (0.37; 12.98 - 14.4)	14.1 (0.36; 13.38 - 14.8)	-0.38 (0.34; 0.2575)
Month 18	13.3 (0.39; 12.5 - 14)	13.9 (0.38; 13.1 - 14.6)	-0.59 (0.37; 0.1131)
Month 21	12.7 (0.4; 11.89 - 13.5)	13.6 (0.39; 12.79 - 14.3)	-0.87 (0.4; 0.0285)
Month 24	12.3 (0.43; 11.49 - 13.2)	12.9 (0.4; 12.12 - 13.7)	-0.59 (0.43; 0.1733)
Month 27	11.6 (0.46; 10.66 - 12.5)	12.9 (0.42; 12.05 - 13.7)	-1.3 (0.48; 0.007)
Month 30	10.8 (0.53; 9.71 - 11.8)	12.2 (0.48; 11.3 - 13.2)	-1.48 (0.59; 0.0126)
Month 33	10.8 (0.58; 9.63 - 11.9)	11.9 (0.51; 10.87 - 12.9)	-1.12 (0.66; 0.0924)
Month 36	10.3 (0.6; 9.12 - 11.5)	11.6 (0.53; 10.52 - 12.6)	-1.26 (0.7; 0.0719)
Month 36 - Baseline	-7.96 (0.54; <0.0001)	-6.17 (0.45; <0.0001)	na

Table S3. LS Means (SE; 95% CI) for Sociability subscale score measured by the Subscale 2 of ATEC. The differences between High- and Low-duration quartiles and between Month 36 and Baseline are presented as: LS Mean (SE; P-value). A lower score indicates lower severity of ASD symptoms. The negative High-Low contrast indicates that the high-duration quartile had lower score and therefore milder symptoms.

Visit Number	High-duration quartile	Low-duration quartile	High-Low
Baseline	15.1 (0.42; 14.29 - 15.9)	14.5 (0.41; 13.67 - 15.3)	0.63 (0.29; 0.0329)
Month 6	14 (0.44; 13.11 - 14.8)	13.9 (0.44; 13.04 - 14.8)	0.07 (0.36; 0.8384)
Month 9	13.7 (0.45; 12.85 - 14.6)	13 (0.44; 12.16 - 13.9)	0.71 (0.37; 0.0567)
Month 12	13.4 (0.47; 12.43 - 14.3)	13.4 (0.46; 12.47 - 14.3)	-0.01 (0.41; 0.9747)
Month 15	12.6 (0.5; 11.64 - 13.6)	13.1 (0.48; 12.13 - 14)	-0.45 (0.47; 0.3367)
Month 18	12.8 (0.52; 11.79 - 13.8)	12.7 (0.51; 11.72 - 13.7)	0.09 (0.52; 0.8678)
Month 21	13 (0.55; 11.91 - 14)	12.8 (0.53; 11.74 - 13.8)	0.21 (0.56; 0.7042)
Month 24	12.5 (0.58; 11.32 - 13.6)	12.5 (0.54; 11.45 - 13.6)	-0.05 (0.6; 0.9296)
Month 27	13 (0.64; 11.75 - 14.3)	13.3 (0.56; 12.16 - 14.4)	-0.26 (0.68; 0.698)
Month 30	12.3 (0.74; 10.87 - 13.8)	11.6 (0.65; 10.34 - 12.9)	0.7 (0.84; 0.4041)
Month 33	12.4 (0.8; 10.87 - 14)	12.4 (0.71; 11.04 - 13.8)	0.03 (0.94; 0.9785)
Month 36	12.8 (0.84; 11.19 - 14.5)	11 (0.74; 9.58 - 12.5)	1.82 (0.99; 0.0663)
Month 36 - Baseline	-2.27 (0.77; 0.0032)	-3.46 (0.65; <0.0001)	na

Table S4. LS Means (SE; 95% CI) for the Sensory/Cognitive Awareness subscale score measured by the Subscale 3 of ATEC. The differences between High- and Low-duration quartiles and between Month 36 and Baseline are presented as: LS Mean (SE; P-value). A lower score indicates lower severity of ASD symptoms. The negative High-Low contrast indicates that the high-duration quartile had lower score and therefore milder symptoms.

Visit Number	High-duration quartile	Low-duration quartile	High-Low
Baseline	16.5 (0.36; 15.7 - 17.2)	16.5 (0.35; 15.8 - 17.2)	-0.04 (0.25; 0.8737)
Month 6	15.8 (0.38; 15.1 - 16.6)	15.5 (0.37; 14.8 - 16.3)	0.26 (0.31; 0.3869)
Month 9	15.5 (0.39; 14.8 - 16.3)	14.8 (0.38; 14 - 15.5)	0.75 (0.32; 0.019)
Month 12	15.1 (0.4; 14.3 - 15.9)	14.9 (0.39; 14.1 - 15.6)	0.19 (0.35; 0.592)
Month 15	14.9 (0.43; 14.1 - 15.7)	14.2 (0.41; 13.4 - 15)	0.72 (0.4; 0.0736)
Month 18	15.1 (0.45; 14.2 - 16)	14.2 (0.43; 13.4 - 15.1)	0.92 (0.44; 0.0383)
Month 21	14.1 (0.47; 13.2 - 15.1)	14 (0.45; 13.2 - 14.9)	0.1 (0.48; 0.828)
Month 24	14.1 (0.5; 13.2 - 15.1)	14.2 (0.46; 13.3 - 15.1)	-0.07 (0.52; 0.8999)
Month 27	13.4 (0.55; 12.3 - 14.5)	14.1 (0.48; 13.1 - 15)	-0.68 (0.58; 0.2384)
Month 30	13.3 (0.63; 12 - 14.5)	13.3 (0.56; 12.2 - 14.4)	-0.02 (0.72; 0.9787)
Month 33	14.2 (0.69; 12.9 - 15.6)	13.3 (0.61; 12.1 - 14.4)	0.96 (0.81; 0.2348)
Month 36	14.2 (0.72; 12.8 - 15.6)	12.6 (0.63; 11.4 - 13.8)	1.58 (0.85; 0.0631)
Month 36 - Baseline	-2.26 (0.66; 0.0006)	-3.89 (0.56; <0.0001)	na

Table S5. LS Means (SE; 95% CI) for Health/Physical/Behavior subscale score measured by the Subscale 4 of ATEC. The differences between High- and Low-duration quartiles and between Month 36 and Baseline are presented as: LS Mean (SE; P-value). A lower score indicates lower severity of ASD symptoms. The negative High-Low contrast indicates that the high-duration quartile had lower score and therefore milder symptoms.

Visit Number	High-duration quartile	Low-duration quartile	High-Low
Baseline	23.1 (0.66; 21.8 - 24.4)	21.5 (0.66; 20.2 - 22.8)	1.63 (0.46; 0.0004)
Month 6	22.2 (0.7; 20.8 - 23.6)	21 (0.7; 19.6 - 22.4)	1.2 (0.56; 0.0315)
Month 9	22.6 (0.71; 21.2 - 24)	20.4 (0.7; 19.1 - 21.8)	2.2 (0.58; 0.0002)
Month 12	22.4 (0.74; 21 - 23.9)	21 (0.72; 19.6 - 22.4)	1.38 (0.63; 0.0297)
Month 15	21.6 (0.78; 20.1 - 23.2)	20.1 (0.75; 18.6 - 21.6)	1.56 (0.72; 0.0311)
Month 18	22.5 (0.82; 20.9 - 24.1)	20.1 (0.8; 18.6 - 21.7)	2.34 (0.8; 0.0035)
Month 21	22.1 (0.85; 20.4 - 23.7)	20.6 (0.82; 19 - 22.2)	1.47 (0.86; 0.0886)
Month 24	21.7 (0.9; 20 - 23.5)	20.5 (0.85; 18.8 - 22.2)	1.24 (0.93; 0.1821)
Month 27	20.9 (0.99; 18.9 - 22.8)	21.6 (0.88; 19.8 - 23.3)	-0.68 (1.04; 0.5123)
Month 30	20.8 (1.14; 18.5 - 23)	19.5 (1.02; 17.5 - 21.5)	1.26 (1.29; 0.3294)
Month 33	23.4 (1.24; 21 - 25.8)	18 (1.1; 15.8 - 20.1)	5.44 (1.44; 0.0002)
Month 36	21.3 (1.3; 18.8 - 23.9)	20.3 (1.14; 18 - 22.5)	1.05 (1.52; 0.4898)
Month 36 - Baseline	-1.78 (1.18; 0.1308)	-1.19 (0.99; 0.2296)	na
